# Unique brood ester profile in a *Varroa destructor* resistant population of European honey bee (*Apis mellifera*)

**DOI:** 10.1038/s41598-024-76399-6

**Published:** 2024-10-26

**Authors:** Nicholas Scaramella, Robert Glinwood, Barbara Locke

**Affiliations:** Department of Ecology, Swedish University of Agricultural Sciences, Uppsala, Sweden

**Keywords:** *Apis mellifera*, *Varroa destructor*, Brood ester pheromones, BEP, Brood effects, Entomology, Ecology, Coevolution

## Abstract

*Varroa destructor* is one of the greatest threats to *Apis mellifera* worldwide and if left untreated will kill a colony in less than three years. A Varroa-resistant population from Gotland, Sweden, has managed to survive for 25 years with little to no Varroa treatment by reducing the mite’s reproductive success. The underlying mechanisms of this trait is currently not known, though previous research indicates that it is the honey bee brood, and not adult bee influence, that contributes to this phenotype. As the mite’s own reproduction is synchronized with the brood’s development though the interception of brood pheromones, it is possible that a change in pheromone profile would disrupt the mite’s reproductive timing. To investigate this, we characterized the brood ester pheromone (BEP) profile of our resistant Gotland population compared to a non-resistant control. This was done by extracting and analyzing key cuticular compounds of the BEP using gas chromatography. A significant difference was found immediately after brood capping, indicating a divergence in their pheromonal production at this time point. This is an important step to understanding the mechanisms of the Gotland population’s Varroa-resistance and contributes to our global understanding of *Varroa destructor* infestation and survival.

## Introduction

The invasive ectoparasitic mite *Varroa destructor* (hereafter referred to as Varroa) is unarguably one of the largest threats to the European honey bee (*Apis mellifera*) causing colony death worldwide^1^. Varroa relies entirely on the honey bee for food and reproduction, which occurs mainly in the cells of developing brood^2^. When Varroa feeds on the honey bee fat bodies and hemolymph, a number of viruses are transmitted to the developing bee pupae, most notably *Deformed Wing Virus* (DWV)^3,4^. DWV causes reduced body weight, a shorter lifespan, and malformed wings resulting in flightless adult bees that cannot contribute to colony functions^5–7^. With exponentially increasing mite infestation vectoring viruses in the brood, a virus epidemic eventually occurs leading to a dwindling adult bee population and ultimately colony mortality within 1–2 years if the mite infestation is not controlled by beekeepers^8–10^. The best defence beekeepers have against high Varroa infestation, and to avoid a virus epidemic, is to use chemical treatments such as synthetic pyrethroids or organic acids such as oxalic or formic acid applied to the hive. These treatments unfortunately can also reduce bee health, and Varroa can develop resistance towards some of these treatments^11,12^. An alternative method towards mitigating the harmful consequences of Varroa infestation is through Varroa resistance selective breeding programs. Several programs, usually focusing on adult bee behaviours that target the mite, such grooming behaviour, hygienic behaviour, and more specifically Varroa Sensitive Hygiene (VSH), where adult bees selectively remove Varroa parasitized brood^13,14^, have had some success in increasing the frequencies of these behaviours but producing long-term stable Varroa resistance has been challenging^15^. A deeper understanding of the complex host-parasite relationship and interactions between Varroa and honey bees is necessary in order to improve the efficacy of Varroa resistance selective breeding programs and increase honey bee resistant stock on a large scale^16–18^.

In the Baltic sea, on the island of Gotland, Sweden, there is a population of honey bees that have survived with Varroa infestation with little to no chemical treatment since 1999^19^. This population exhibits naturally adapted Varroa-resistant phenotypes, specifically the ability to reduce mite reproductive success rates. Only around 50% of the mother mites in the Gotland Varroa-resistant population are able to produce viable offspring at a given occasion^20,21^ compared to non-resistant regularly managed honey bee colonies, where mother mites have reproductive success rates over 80%^20,21^. While it is still unclear how the bees reduce the mite reproduction, it is clearly a genetic feature of the bee population, rather than a reduction of virulence on the side of the mite^22,23^ and has been a stable trait in the population observed over multiple occasions since it was first reported in 2011^20,24,25^. Recent research shows that the reduced mite reproduction, in this and other naturally adapted mite resistant honey bee populations, appears to be due to characteristics of the honey bee brood, as opposed to Varroa resistance behaviours of adult worker bees^24^, which are often the focus in Varroa resistance breeding programs. This population therefore, provides a unique opportunity to study the natural relationship and interactions between Varroa mites and European honey bees.

Chemical signaling is a major method of communication between developing brood and attending nurse bees within a honey bee colony^26,27^. In particular, a cocktail of different volatile compounds have been identified in the brood ester pheromone (BEP) profile of the brood that are used to communicate information to adult bees such as the brood’s caste or age^27–29^. Originally ten BEP compounds have been identified in brood communication: fatty acid methyl (FAME) & ethyl esters (FAEE) of palmitate, linoleate, stearate, oleate, and linolenate acids, with E-β-Ocimene, a terpene, discovered later^28,30^. These BEP compounds are known to cause changes in the behavior and biology of the receiving nurse bees depending on the timing and amount of BEP’s produced^26,31^. The effects of the specific BEP compounds can vary and range from methyl palmitate & methyl linolenate initiating capping to ethyl palmitate & methyl linolenate preventing ovary development in worker bees, among other effects^27,29,32,33^.

These BEP compounds can be classified as kairomones, instead of pheromones, when they are intercepted by unintended target organisms such as ectoparasites like Varroa. The same BEP compounds that the brood produce to communicate with worker bees, such as methyl linoleate and ethyl palmitate, are intercepted by Varroa as signals on the timing for brood cell invasion^34–38^. Variation in BEP profiles exists between the different castes within a honey bee colony, and this affects the ability of Varroa to exploit them. For example, Varroa is often more attracted to drone brood as they produce a larger quantity of BEP compounds over a longer period of time compared to worker brood^39,40^. The BEP profile is also a major factor in the large reduction in Varroa infestation of queen cells as queen brood produce larger amounts of methyl oleate, which is a Varroa repellent^37^.

Mite reproduction is tightly synchronized to brood development, with mite oogenesis linked to certain BEP volatiles produced by the brood at specific times^41,42^.The first 12 h post capping of the brood cell are critical for mite reproductive success. A disruption in the BEP communication between the developing pupae and the mite during this time can cause the invading mite (foundress) to reabsorb any eggs that she has started to produce^41,43^. Therefore, even slight alterations in the brood’s BEP profile could break the kairomone-timing network and lead to reduction in successful mite reproduction.

The aim of this study was to characterize the BEP profile of developing brood in the Varroa resistant population from Gotland, Sweden over time and identify any possible alterations of the BEP profile that could explain the reduced mite reproductive success observed in this population. This was approached by comparing the timing and quantity of cuticle volatiles produced by brood collected from the Varroa resistant honey bee population on Gotland, Sweden (hereafter referred to as resistant honey bees) with a control population of non-resistant honey bees. Cuticular volatiles were chemically extracted at biologically relevant time points during the early stages of the post-capping period when mite reproduction is initiated and were identified and quantified using gas chromatography (GC). We hypothesized that if a change in the BEP profile is responsible for the reduced mite reproduction phenotype in this resistant honey bee population, we would see a significant difference in the timing or quantity of the BEP profile produced between the two populations.

## Methods

Twelve experimental honey bee colonies were established during June of 2021 from splitting six non-resistant honey bee colonies equally. Non-resistant colonies were purchased from a private beekeeper on Åland, Finland. Half of the colonies kept their original non-resistant queens and became the control group for this experiment, while the other six colonies were given mated queens obtained from the mite-resistant population located on Gotland, Sweden^20^. The colonies were given a minimum of four weeks to allow a replacement of the brood so that any larvae in the colony at the time of sampling were known to be produced by the resistant or non-resistant, control group queen. All experimental colonies were located in a single apiary at the Swedish University of Agricultural Sciences (GPS Coordinates: 59° 48′ 55.60596″, 17° 39′ 54.39866″) and managed with normal beekeeping practices with the exception that no Varroa control treatment was performed.

Eight-day old larvae were checked hourly to capture the time point when the brood cell was being capped. Using a transparent acetate sheet overlay on the frame of brood, cells that were newly capped were marked out as the 0 h for our experiment. Individual pupae were extracted from their brood cells over a time-series at 00, 06, 12, 18, 24, and 36 h post capping using the transparent acetate sheet overlay to identify the post-capping age of individual brood cells.

To extract the BEP volatile compounds, frames were removed from their respective hives and transferred to a designated indoor workspace. Using forceps, the cell capping was opened, and the developing pupae was careful removed. The pupae were placed on filter paper (Munktell’s Swedish Filter Paper; No. 8, 9 cm) to ensure that their cuticle had not been punctured during removal and to locate any Varroa infestation. Pupae with a cuticle puncture or Varroa infestation were excluded from the experiment. Forceps were flamed between each colony and time point to minimize cross contamination of volatiles compounds.

Each chemical sample contained 4 pupae pooled together for each time point per colony. The pupae were submerged in 2 ml (1.25g) of n-pentane for 10 min, following the procedure detailed in Frey et al.^41^. Chemical extracts were stored in glass vials (Thermo Scientific 1.1 ml screw top tapered glass vials) and immediately put in a – 20 °C freezer before being transferred to a   − 80 °C freezer.

For sample concentration, vials were removed from the freezer and left at room temperature for 5 min to thaw completely. They were then agitated for 15 s to homogenize the mixture before being concentrated to 100 μl under a gentle nitrogen flow.

For tentative identification of target compounds, samples were analyzed by gas chromatography-mass spectrometry (GC/MS) on an Agilent 7890N (Agilent Technologies) GC coupled to an Agilent 5975C mass selective detector (electron impact 70 eV). The GC was equipped with an HP-1 column (100% dimethyl polysiloxane, 50 m, 0.32 mm i.d. and 0.52 μm film thickness, J&W Scientific, USA), and fitted with a cold on column inlet. The GC temperature program was 30°C/4 min, 5°C/min to 150°C/0.1 min, 10°C/min to 250°C/15 min, using helium as carrier with a flow rate of 1.3 ml/min. BEPs present in the samples were identified by comparison against a commercially available library (NIST 08) and by comparison of mass spectra and retention indices with commercially available authentic standards (Sigma-Aldrich, Sweden). Based on the above analyses, the following compounds were selected for quantification: FAMEs methyl palmitate (Methyl hexadecanoate/MP), methyl linoleate (methyl (9Z,12Z)-octadeca-9,12-dienoate/ML) and methyl stearate (Methyl octadecanoate/MS), FAEEs ethyl palmitate (Ethyl hexadecanoate/EP), ethyl linoleate ((9Z,12Z,15Z)-Ethyl octadeca-9,12,15-trienoate/EL), ethyl stearate (Ethyl octadecanoate/ES), and the monoterpene, (*E*)-β-ocimene (EO).

For quantification, the concentrated samples (2 µl injections) were analyzed by gas chromatography (GC) on an Agilent 6890N with a flame ionization detector equipped with an HP-1 column (100% dimethylpolysiloxane, 50 m, 0.32 mm i.d., 0.52 μm film thickness, J&W Scientific, Folsom, CA), with hydrogen as carrier gas and fitted with a cold on column inlet.

The GC temperature program was 40°C for 1 min, 10°C/min to 280°C and held at 280°C for 10 min.

The amount of each target compound was calculated relative to the FID response to commercially available authentic standards (Merck, Sweden; 1 µl injection of 5 ng/µl standard solution).

### Statistical analysis

Statistical analyses were performed using R version 4.0.1 and R Studio Version 1.3.959 using the R packages “lme4” (version 1.1.35.1), “car” (v. 3.1.2), “moments” (v. 0.14.1), “glmmTMB” (v. 1.1.8), “DHARMa” (v. 0.4.6), “performance” (v. 0.11.0), “RVAideMemoire” (v. 0.9.83.7), “emmeans” (v. 1.10.0), “effects” (v. 4.2.2), and “bestNormalize” (v. 1.9.1)^44,45^ with all graphs made using the R package “tidyverse” (version 2.0.0).

A generalized mixed effect model was used to compare BEP differences between backgrounds. Individual models were used for each compound analyzed as well as the combined FAME and FAEE’s results. The BEP compound quantities were used as a response variable, with background and time points used as fixed variables, and hive origin as a random variable. Zero inflation adjustment was performed on all models. Combined FAME, Methyl & Ethyl Linoloate, and (E)-β-Ocimene were square root transformed while combined FAEE, Methyl & Ethyl Palmitate, and Methyl & Ethyl Stearate were arcsign transformed to improve model fit. An estimated marginal means (emmeans) post-hoc pairwise comparison of the generalized mixed effect model was done for comparing the effect of background on compound levels within each time point, as well as with one step forward in time (i.e. 00H vs 006H).

## Results

**Fig. 1:**
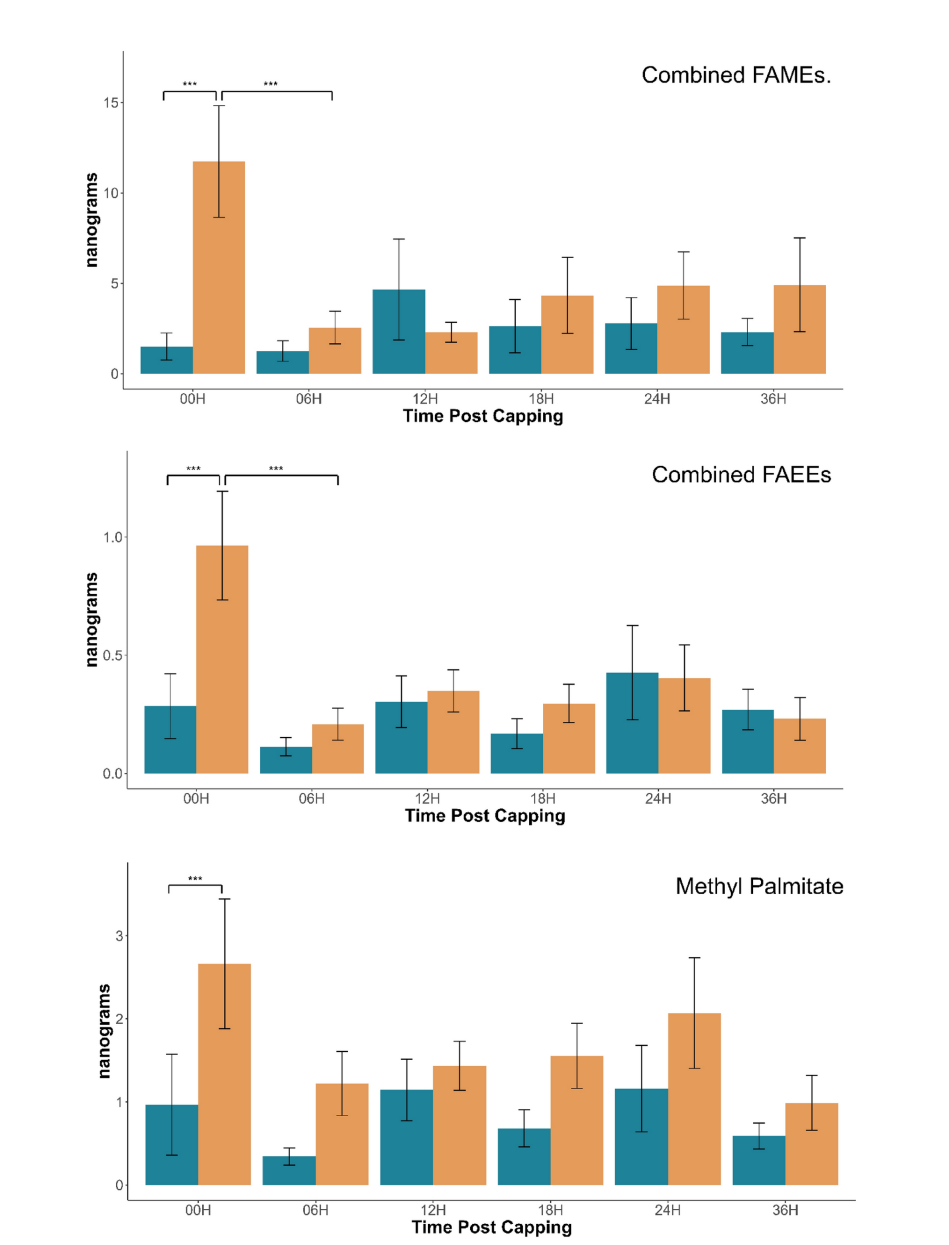

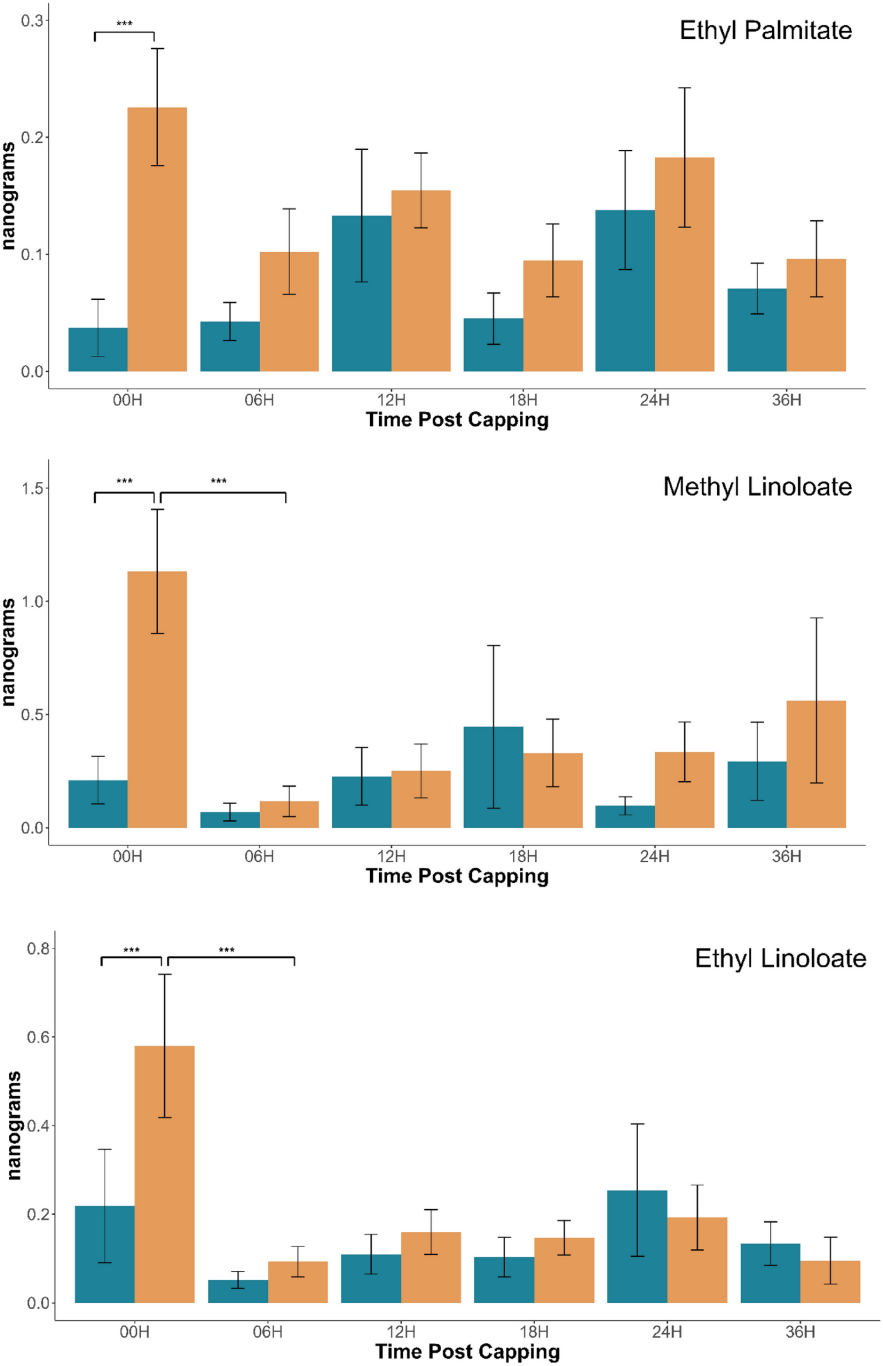

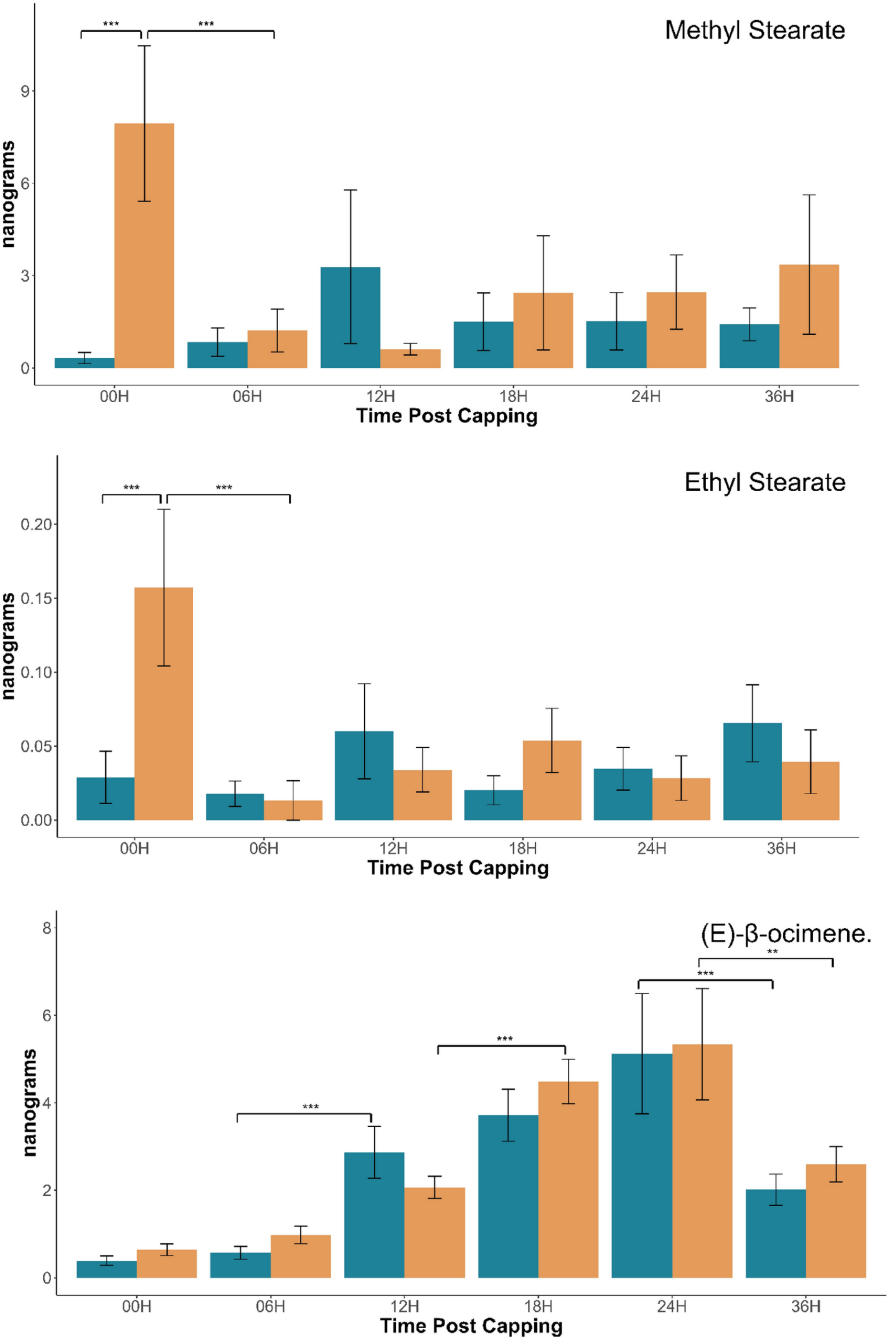
Amount of named compound present on the cuticle of *Apis mellifera* worker brood 00, 06, 12, 18, 24, and 36 h after cell capping. Blue = Resistant, Orange = Non-Resistant. n = 18 (Resistant 00H, 06H, 12H, 18H, 36H; Non-Resistant 00H); n = 17 (Resistant 24H; Non-Resistant 06H, 12H, 18H, 24H, 36H) p value of significant differences added. Errors bars representing standard error used.

For all BEPs measured, we found lower amounts in the resistant population at almost all time points compared to the control population (Fig. 1, Table 1). While only the 00H time point was significantly different between our populations across all BEPs, we see a clear trend that lower amounts of BEP were produced by the resistant population compared to the control population in 38 out of 46 comparisons (83%). The main exception to this trend appears to be the production of methyl stearate at the 12H time point, which interestingly is also the compound with the most dramatic difference at the 00H time point (p < 0.001; Fig. 1).

**Table 1:** Results of generalized mixed effect model.

	Chi Sq	df	p
A. Combined FAMEs			
Intercept	123.499	1	** < 0.005**
Background	19.476	1	** < 0.005**
Time	21.884	5	** < 0.005**
Back*Time	12.420	5	**0.029**
B. Combined FAEE			
Intercept	72.768	1	** < 0.005**
Background	14.519	1	** < 0.005**
Time	19.291	5	** < 0.005**
Back*Time	10.578	5	0.060
C. Methyl Palmitate			
Intercept	59.015	1	** < 0.005**
Background	14.562	1	** < 0.005**
Time	11.203	5	**0.047**
Back*Time	6.219	5	0.285
D. Ethyl Palmitate			
Intercept	38.849	1	** < 0.005**
Background	13.512	1	** < 0.005**
Time	10.535	5	0.061
Back*Time	7.165	5	0.208
E. Methyl Linoloate			
Intercept	59.205	1	** < 0.005**
Background	15.806	1	** < 0.005**
Time	22.693	5	** < 0.005**
Back*Time	10.024	5	0.075
F. Ethyl Linoloate			
Intercept	72.124	1	** < 0.005**
Background	12.658	1	** < 0.005**
Time	24.873	5	0.061
Back*Time	10.839	5	0.055
G. Methyl Stearate			
Intercept	84.552	1	** < 0.005**
Background	25.176	1	** < 0.005**
Time	28.960	5	** < 0.005**
Back*Time	21.992	5	** < 0.005**
H. Ethyl Stearate			
Intercept	43.436	1	** < 0.005**
Background	15.532	1	** < 0.005**
Time	25.677	5	** < 0.005**
Back*Time	16.508	5	** < 0.005**
I. (E)-β-Ocimene			
Intercept	84.552	1	** < 0.005**
Background	25.176	1	0.267
Time	28.960	5	** < 0.005**
Back*Time	21.992	5	0.581

Chemical in table title used as response variable. Background and time used as fixed variable. Hive was used as a random variable. Zero inflation adjustment was performed on all models. FAME, Methyl & Ethyl Linoloate, E-Ocimene were square root transformed to improve model fit. FAEE, Methyl & Ethyl Palmitate, Methyl & Ethyl Stearate were arcsign transformed to improve model fit. Significant values in bold. FAME & FAEE values calculated by adding all Methyl (FAME) or Ethyl (FAEE) values.

Colony background was a significant factor for all chemicals, excluding EO (p < 0.005), indicating that the resistant Gotland bees have a unique overall BEP profile in the first 36 h post-capping when compared to the non-resistant population, characterized by the overall lower BEP production throughout. Time was also a significant factor for MP (p = 0.047), ML (p < 0.005), MS (p < 0.005), ES (p < 0.005), and EO (p < 0.005) with our non-resistant population having a much higher production of the stated chemicals at 0H before a significant decrease at the 6H mark, with the exception of EO which had a steady increase over time before decreasing at the 36H mark (Fig. 1, Table 1, Supplemental Table S1). The interaction between background and time was significant only for MS (p < 0.005) and ES (p < 0.005), with EL falling just short of significant (p = 0.055) (Fig. 1, Table 1) (Fig. 1, Supplemental Table S1).

The most significant differences between the populations occurred at 00H, with lower amounts of all BEP analyzed in the resistant population (p = 0.0029 (MP); 0.0049 (EP); 0.0008 (ML); 0.0038 (EL); < 0.0001 (MS); 0.0009 (ES)) (Fig. 1, Supplemental Table S1). This is continued by a non-significant trend throughout all time points of less compounds produced by the resistant colonies. For the non-resistant population there is a significant drop for ML (p = 0.0003), EL (p = 0.0006), MS (p = 0.0018), and ES (p = 0.0003) between 00 and 06H, returning to non-significant differences at further time points (Fig. 1, Supplemental Table S1). One exception is EO, where we instead see a steady increase until a significant drop in both populations at 36H.

## Discussion

This study demonstrates biologically important differences in brood ester pheromones (BEP’s) in a unique Varroa resistant population, compared to non-resistant control population, produced at time points during pupal development that are fundamentally relevant to disrupting Varroa mite reproduction. Specifically, a significant difference was observed between the two populations at the 00H time point just after the larvae are capped in their cells for pupation. Overall lower amounts of BEP were produced in the resistant population, which suggests a type of chemical camouflage, more specifically what we have termed chemical whispering to disrupt or interfere with the initiation of mite reproduction. Many of these compounds have been found to be Varroa attractants as well as to initiate mite reproduction^34–38^. By reducing the overall BEP produced, the signal may be more difficult for the mite to intercept, while still being recognizable by adults with increased sensitivity to chemical recognition^46^.

Frey et al.^41^ found that if pupal BEP volatiles were artificially added to brood cells 24 h after capping (well after the 12 h critical period), there was a significant increase in mite reproduction. While the exact chemicals used by the mite to initiate oogenesis are still unknown, there is clear evidence that Varroa use BEP compounds as instigators for reproduction^37,38,41^. Frey further found a decline in BEP production around the critical 12 h post capping time point, suggesting these BEPs as possible candidates as Varroa reproductive kairomones. While the authors were not able to say that the two occurrences are linked, FAEE’s may be involved in the initial activation of Varroa reproduction^41^. This could be a possible explanation to the observed lower amounts of BEP in our resistant populations.

Previous research with high resolution QTL analysis on the resistant honey bee population on Gotland found three genes relating to the Varroa resistance phenotype of reduced mite reproduction; Phantom, Cyp18a1, and Mblk-1^47^. While these genes are not directly linked to BEP production they are significant for brood health and development by initiating metamorphosis and molting through the ecdysone biosynthesis pathway^47–53^. This means they are all active during the critical mite reproduction time-period. However, the authors note that much of the resistant phenotype variation still remains unexplained by these genes^47^. Gene expression analysis of the BEP biosynthesis pathways, as performed by Qin et al. may be beneficial to understand not only the possible genetic components of the observed differences, but also the mechanisms that create differences in the final BEP products we observed in this study.

In order for a pheromone change to persist in a population there must not only be a different pheromonal profile created by the signaller (in this case, the brood), but also for it to be received and interpreted correctly by the receiver (in this case, the nurse bees). Theoretical modelling of the coevolution between signallers and receivers has two major predictions, that (1) the selective pressure of receivers should be greater than those on the signaller^54^ and (2) when chemical communication is under strong selective pressure, natural selection should favour receivers that are able to detect a wide range of novel compounds as well as novel ratios of compounds^55^. Based on these predictions, if a signaler alters their BEP profile, a receiver theoretically should be able to adapt and correctly interpret the new signal, particularly in a system with strong selective pressure. Varroa represents a strong selective pressure towards its host with an exponential population growth rate and by vectoring viruses that lead to colony death. It is therefore not unlikely that adaptations on chemical communication in this population could have resulted in a short timeframe. While these arguments of “receiver advantage” can also be applied to Varroa’s receiving of kairomones, this may be compounded with its lower genetic diversity and high occurrence of inbreeding compared to the honey bee^56–61^.

In classic host-parasite co-evolution theory, the parasite is usually viewed as having the “advantage” in an arms race due to their shorter generation times and larger population size leading to more rapid adaptations than their host^62–64^. A rare advantage that the honey bee brood may have over Varroa however is that the unintended receiver of an olfactory signal, like the intended received, must be able to detect the specific compounds of the signal as well as be able to interpret them correctly^65,66^. While we know that Varroa possess the receptors necessary to intercept the broods signals^67^, it has also been suggested that with minor changes in the emitter’s genetics, new pheromone compounds and blends can be produced^55,68–72^. If this genetic variation pre-existed in the population, then the evolutionary response to parasitism may occur quite rapidly, in some cases only taking a handful of generations^73–75^. The possibility of rapid changes in pheromonal signals, as mentioned above, could result in the mite having increased difficulty in adapting to the shifting signals.

In predator–prey systems, where kairomones are intercepted by predator species, there are examples of prey changing their pheromonal composition to camouflage themselves over a relatively short ecological time frame^2^. Over just three years bark beetles (*Ips pini*) altered the blend of their pheromonal compounds between the preferences of two predators, as well as incorporating a synergistic compound that increased the receptiveness of conspecifics with no additional reaction from predators^2^. Similarly, the honey bee population on Gotland has been naturally exposed to uncontrolled Varroa infestation and displayed unique resistance phenotypes after only a short time. In parasite and parasitoid systems we can also see a reliance on cuticular kairomones by the parasite/parasitoids for information related to reproductive conditions that if disrupted may increase difficulties in finding hosts or spatial/temporal optimums^76–81^. This would reduce reproductive success and may be similar to what we are seeing in our own host/parasite system.

Further research is needed to determine how the differences in BEP between our resistant and non-resistant populations observed in this study have an effect on Varroa reproduction, and if there are trade-offs on the overall health and survival of these resistant colonies with these differences in BEP for the communication between brood and adult. Across other studies there is large variation in quantities, timings, and ratios of BEP profiles, making comparisons between different experiments difficult and raising questions on what a typical BEP profile is, or if one even exists^31,35,38,41,82^. While our study was designed to reduce temporal, spatial and methodological variation, we cannot be sure that the BEP profiles in the non-resistant honeybee can be considered standard. A large-scale study looking at brood BEP profiles across time, space, and genetics with standardized methodology would help to better understand what should be considered typical or atypical and would help when comparing populations in vastly different environments as well as to create a better understanding of brood development and capping signals. Further, in this experiment, only non-infested brood were collected in order determine a baseline BEP profile of our populations without interference of Varroa presence. An important consideration for future work would be to examine the plasticity of the production of BEP in response to Varroa mite infestation to determine if, and how, BEP profiles differ when larvae are infested. A behavioural assay on Varroa mite choice for host selection, using similar methods to either Pernal et al.^83^ or Li et al.^84^, would also help provide an understanding of the mite’s reproductive preferences. Coating live or dummy larvae with increased levels of the BEPs found to be reduced in the resistance larvae of this study, would help to characterize their role in both Varroa mite host selection and Varroa reproductive success. Finally, gene expression and proteomic analysis would contribute to a more complete understanding of what is happening with the brood during these critical time points of mite reproduction.

In conclusion, this study has demonstrated clear differences of BEP production at biologically relevant time points in the brood of a Varroa resistant honeybee population compared with a non-resistant population and provides a strategic foundation for future research looking at honey bee adaptations towards Varroa mite infestation and the evolution of this unique host-parasite system.

## Supplementary Information

Supplementary Information.

## Data Availability

The datasets used and/or analysed during the current study available from the corresponding author (nicholas.scaramella@slu.se) on reasonable request. The dataset will also be stored in the Swedish National Data Service repository, [https://doi.org/10.5878/h2hc-h513].
