# Supplementary material for: Unique brood ester profile in a Varroa destructor resistant population of European honey bee (Apis mellifera)
Source: Sci Rep. 2024 Oct 26;14:25531. doi: 10.1038/s41598-024-76399-6 (PMC11513966; doi:10.1038/s41598-024-76399-6)
Supplement: Supplementary file 1 — Supplementary Information. [file 41598_2024_76399_MOESM1_ESM.docx]

Supplemental Information

**Supplemental Table1.** Results of emmeans post-hoc pairwise comparison of model used in Table 1 between populations for combined FAME, FAEE, and individual chemicals at each time point. Background*Time used for comparison. Significant values in bold. CTL, Control; RES, Resistant. 0, 6, 12 , 18 , 24, & 36h = 0, 6, 12, 18, 24, 36 hours post capping.

1. Combined FAME

|  | estimate | SE | df | t ratio | p value |
| --- | --- | --- | --- | --- | --- |
| RESxNRES-00H | - 2.0353 | 0.458 | 195 | -4.444 | **0.0002** |
| RES-00Hx06H | 0.0488 | 0.490 | 195 | 0.100 | 0.9673 |
| NRES-00Hx06H | 1.5649 | 0.429 | 195 | 3.644 | **0.0028** |
| RESxNRES-06H | -0.5192 | 0.467 | 195 | -1.112 | 0.9673 |
| RES-06Hx12H | -0.3904 | 0.453 | 195 | -0.862 | 0.9673 |
| NRES-06Hx12H | 0.0640 | 0.429 | 195 | 0.149 | 0.9673 |
| RESxNRE-12H | -0.0648 | 0.413 | 195 | -0.157 | 0.9673 |
| RES-12Hx18H | -0.0195 | 0.475 | 195 | -0.041 | 0.9673 |
| NRES-12Hx18H | -0.3280 | 0.421 | 195 | -0.779 | 0.9673 |
| RESxNRES-18H | -0.3732 | 0.482 | 195 | -0.774 | 0.9673 |
| RES-18Hx24H | -0.0755 | 0.500 | 195 | -0.151 | 0.9673 |
| NRES-18Hx24H | -0.2379 | 0.447 | 195 | -0.533 | 0.9673 |
| RESxNRES-24H | -0.5356 | 0.468 | 195 | -1.145 | 0.9673 |
| RES-24Hx36H | -0.0688 | 0.492 | 195 | -0.140 | 0.9673 |
| NRES-24Hx36H | 0.2444 | 0.480 | 195 | 0.509 | 0.9673 |
| RESxNRES-36H | 0.2225 | 0.503 | 195 | 0.442 | 0.9673 |

1. Combined FAEE

|  | estimate | SE | df | t ratio | p value |
| --- | --- | --- | --- | --- | --- |
| RESxNRES-00H | -0.5131 | 0.135 | 195 | -3.810 | **0.0018** |
| RES-00Hx06H | 0.0858 | 0.135 | 195 | 0.637 | 0.7065 |
| NRES-00Hx06H | 0.5128 | 0.137 | 195 | 3.754 | **0.0018** |
| RESxNRES-06H | -0.0860 | 0.137 | 195 | -0.629 | 0.7065 |
| RES-06Hx12H | -0.1291 | 0.135 | 195 | -0.958 | 0.7065 |
| NRES-06Hx12H | -0.1700 | 0.139 | 195 | -1.227 | 0.7065 |
| RESxNRE-12H | -0.1269 | 0.137 | 195 | -0.929 | 0.7065 |
| RES-12Hx18H | 0.0859 | 0.135 | 195 | 0.638 | 0.7065 |
| NRES-12Hx18H | 0.0707 | 0.139 | 195 | 0.510 | 0.7513 |
| RESxNRES-18H | -0.1421 | 0.137 | 195 | -1.040 | 0.7065 |
| RES-18Hx24H | -0.1838 | 0.137 | 195 | -1.346 | 0.7065 |
| NRES-18Hx24H | -0.0507 | 0.139 | 195 | -0.366 | 0.7897 |
| RESxNRES-24H | -0.0089 | 0.139 | 195 | -0.064 | 0.9488 |
| RES-24Hx36H | 0.0901 | 0.137 | 195 | 0.659 | 0.7065 |
| NRES-24Hx36H | 0.1443 | 0.139 | 195 | 1.042 | 0.7065 |
| RESxNRES-36H | -0.0453 | 0.137 | 195 | -0.332 | 0.7897 |

1. Methyl Palmitate

|  | estimate | SE | df | t ratio | p value |
| --- | --- | --- | --- | --- | --- |
| RESxNRES-00H | -1.079 | 0.283 | 195 | -3.816 | **0.0029** |
| RES-00Hx06H | 0.186 | 0.266 | 195 | 0.699 | 0.5916 |
| NRES-00Hx06H | 0.720 | 0.287 | 195 | 2.507 | 0.1039 |
| RESxNRES-06H | -0.545 | 0.273 | 195 | -1.998 | 0.1963 |
| RES-06Hx12H | -0.450 | 0.260 | 195 | -1.730 | 0.2219 |
| NRES-06Hx12H | -0.179 | 0.276 | 195 | -0.648 | 0.5916 |
| RESxNRE-12H | -0.274 | 0.264 | 195 | -1.035 | 0.5916 |
| RES-12Hx18H | 0.192 | 0.270 | 195 | 0.712 | 0.5916 |
| NRES-12Hx18H | -0.106 | 0.290 | 195 | -0.366 | 0.7149 |
| RESxNRES-18H | -0.572 | 0.289 | 195 | -1.980 | 0.1963 |
| RES-18Hx24H | -0.191 | 0.281 | 195 | -0.680 | 0.5916 |
| NRES-18Hx24H | -0.132 | 0.312 | 195 | -0.422 | 0.7149 |
| RESxNRES-24H | -0.513 | 0.308 | 195 | -1.667 | 0.2219 |
| RES-24Hx36H | 0.238 | 0.276 | 195 | 0.862 | 0.5916 |
| NRES-24Hx36H | 0.547 | 0.307 | 195 | 1.782 | 0.2219 |
| RESxNRES-36H | 0.204 | 0.270 | 195 | 0.755 | 0.5916 |

1. Ethyl Palmitate

|  | estimate | SE | df | t ratio | p value |
| --- | --- | --- | --- | --- | --- |
| RESxNRES-00H | -0.183 | 0.050 | 195 | -3.677 | **0.0049** |
| RES-00Hx06H | -0.006 | 0.050 | 195 | -0.122 | 0.9029 |
| NRES-00Hx06H | 0.119 | 0.051 | 195 | 2.354 | 0.1566 |
| RESxNRES-06H | -0.058 | 0.051 | 195 | -1.150 | 0.4026 |
| RES-06Hx12H | -0.084 | 0.050 | 195 | -1.693 | 0.2699 |
| NRES-06Hx12H | -0.052 | 0.051 | 195 | -1.021 | 0.4433 |
| RESxNRE-12H | -0.026 | 0.051 | 195 | -0.516 | 0.6687 |
| RES-12Hx18H | 0.082 | 0.050 | 195 | 1.649 | 0.2699 |
| NRES-12Hx18H | 0.059 | 0.051 | 195 | 1.153 | 0.4026 |
| RESxNRES-18H | -0.049 | 0.051 | 195 | -0.972 | 0.4433 |
| RES-18Hx24H | -0.088 | 0.051 | 195 | -1.743 | 0.2699 |
| NRES-18Hx24H | -0.082 | 0.051 | 195 | -1.592 | 0.2699 |
| RESxNRES-24H | -0.043 | 0.051 | 195 | -0.832 | 0.5005 |
| RES-24Hx36H | 0.062 | 0.051 | 195 | 1.236 | 0.4026 |
| NRES-24Hx36H | 0.080 | 0.051 | 195 | 1.570 | 0.2699 |
| RESxNRES-36H | 0.025 | 0.051 | 195 | 0.487 | 0.6687 |

1. Methyl Linoleate

|  | estimate | SE | df | t ratio | p value |
| --- | --- | --- | --- | --- | --- |
| RESxNRES-00H | -0.616 | 0.155 | 195 | -3.976 | **0.0008** |
| RES-00Hx06H | 0.110 | 0.155 | 195 | 0.709 | 0.8795 |
| NRES-00Hx06H | 0.691 | 0.157 | 195 | 4.391 | **0.0003** |
| RESxNRES-06H | -0.036 | 0.157 | 195 | -0.225 | 0.9393 |
| RES-06Hx12H | -0.092 | 0.155 | 195 | -0.593 | 0.8795 |
| NRES-06Hx12H | -0.131 | 0.160 | 195 | -0.823 | 0.8795 |
| RESxNRE-12H | -0.075 | 0.157 | 195 | -0.476 | 0.8795 |
| RES-12Hx18H | -0.078 | 0.155 | 195 | -0.505 | 0.8795 |
| NRES-12Hx18H | -0.061 | 0.160 | 195 | -0.382 | 0.8795 |
| RESxNRES-18H | -0.057 | 0.157 | 195 | -0.366 | 0.8795 |
| RES-18Hx24H | 0.112 | 0.157 | 195 | 0.711 | 0.8795 |
| NRES-18Hx24H | -0.022 | 0.160 | 195 | -0.135 | 0.9490 |
| RESxNRES-24H | -0.191 | 0.160 | 195 | -1.197 | 0.8795 |
| RES-24Hx36H | -0.072 | 0.157 | 195 | -0.456 | 0.8795 |
| NRES-24Hx36H | 0.010 | 0.160 | 195 | 0.064 | 0.9490 |
| RESxNRES-36H | 0.109 | 0.157 | 195 | 0.693 | 0.8795 |

1. Ethyl Linoleate

|  | estimate | SE | df | t ratio | p value |
| --- | --- | --- | --- | --- | --- |
| RESxNRES-00H | -0.363 | 0.102 | 195 | -3.558 | **0.0038** |
| RES-00Hx06H | 0.113 | 0.102 | 195 | 1.101 | 0.7264 |
| NRES-00Hx06H | 0.437 | 0.104 | 195 | 4.210 | **0.0006** |
| RESxNRES-06H | -0.040 | 0.104 | 195 | -0.381 | 0.9377 |
| RES-06Hx12H | -0.056 | 0.102 | 195 | -0.544 | 0.8543 |
| NRES-06Hx12H | -0.118 | 0.105 | 195 | -1.121 | 0.7264 |
| RESxNRE-12H | -0.102 | 0.104 | 195 | -0.982 | 0.7479 |
| RES-12Hx18H | -0.003 | 0.102 | 195 | -0.031 | 0.9752 |
| NRES-12Hx18H | 0.012 | 0.105 | 195 | 0.112 | 0.9752 |
| RESxNRES-18H | -0.087 | 0.104 | 195 | -0.838 | 0.7571 |
| RES-18Hx24H | -0.119 | 0.104 | 195 | -1.147 | 0.7264 |
| NRES-18Hx24H | -0.025 | 0.105 | 195 | -0.242 | 0.9752 |
| RESxNRES-24H | 0.007 | 0.105 | 195 | 0.063 | 0.9752 |
| RES-24Hx36H | 0.075 | 0.104 | 195 | 0.719 | 0.7571 |
| NRES-24Hx36H | 0.145 | 0.105 | 195 | 1.375 | 0.7264 |
| RESxNRES-36H | -0.077 | 0.104 | 195 | -0.739 | 0.7571 |

1. Methyl Stearate

|  | estimate | SE | df | t ratio | p value |
| --- | --- | --- | --- | --- | --- |
| RESxNRES-00H | -2.110 | 0.421 | 195 | -5.018 | **<0.0001** |
| RES-00Hx06H | -0.259 | 0.440 | 195 | -0.589 | 0.8177 |
| NRES-00Hx06H | 1.610 | 0.428 | 195 | 3.757 | **0.0018** |
| RESxNRES-06H | -0.242 | 0.447 | 195 | -0.541 | 0.8177 |
| RES-06Hx12H | -0.544 | 0.564 | 195 | -0.964 | 0.8177 |
| NRES-06Hx12H | 0.282 | 0.431 | 195 | 0.653 | 0.8177 |
| RESxNRE-12H | 0.584 | 0.555 | 195 | 1.052 | 0.8177 |
| RES-12Hx18H | 0.249 | 0.574 | 195 | 0.435 | 0.8177 |
| NRES-12Hx18H | -0.307 | 0.412 | 195 | -0.745 | 0.8177 |
| RESxNRES-18H | 0.028 | 0.447 | 195 | 0.061 | 0.9511 |
| RES-18Hx24H | 0.078 | 0.453 | 195 | 0.173 | 0.9204 |
| NRES-18Hx24H | -0.405 | 0.445 | 195 | -0.910 | 0.8177 |
| RESxNRES-24H | -0.456 | 0.452 | 195 | -1.010 | 0.8177 |
| RES-24Hx36H | -0.225 | 0.452 | 195 | -0.498 | 0.8177 |
| NRES-24Hx36H | 0.327 | 0.458 | 195 | 0.713 | 0.8177 |
| RESxNRES-36H | -0.098 | 0.458 | 195 | -0.209 | 0.9204 |

1. Ethyl Stearate

|  | estimate | SE | df | t ratio | p value |
| --- | --- | --- | --- | --- | --- |
| RESxNRES-00H | -0.122 | 0.0311 | 195 | -3.941 | **0.0009** |
| RES-00Hx06H | 0.011 | 0.0311 | 195 | 0.352 | 0.8290 |
| NRES-00Hx06H | 0.139 | 0.0315 | 195 | 4.413 | **0.0003** |
| RESxNRES-06H | 0.006 | 0.0315 | 195 | 0.183 | 0.8546 |
| RES-06Hx12H | -0.041 | 0.0311 | 195 | -1.321 | 0.7554 |
| NRES-06Hx12H | -0.021 | 0.0320 | 195 | -0.646 | 0.7554 |
| RESxNRE-12H | 0.026 | 0.0315 | 195 | 0.831 | 0.7554 |
| RES-12Hx18H | 0.039 | 0.0311 | 195 | 1.247 | 0.7554 |
| NRES-12Hx18H | -0.022 | 0.0320 | 195 | -0.683 | 0.7554 |
| RESxNRES-18H | -0.034 | 0.0315 | 195 | -1.091 | 0.7554 |
| RES-18Hx24H | -0.013 | 0.0315 | 195 | -0.420 | 0.8290 |
| NRES-18Hx24H | 0.027 | 0.0320 | 195 | 0.857 | 0.7554 |
| RESxNRES-24H | 0.006 | 0.0320 | 195 | 0.196 | 0.8546 |
| RES-24Hx36H | -0.032 | 0.0315 | 195 | -1.003 | 0.7554 |
| NRES-24Hx36H | -0.013 | 0.0320 | 195 | -0.408 | 0.8290 |
| RESxNRES-36H | -0.025 | 0.0315 | 195 | -0.787 | 0.7554 |

1. (E)-β-ocimene

|  | estimate | SE | df | t ratio | p value |
| --- | --- | --- | --- | --- | --- |
| RESxNRES-00H | -0.223 | 0.201 | 195 | -1.109 | 0.5101 |
| RES-00Hx06H | -0.113 | 0.202 | 195 | -0.558 | 0.6628 |
| NRES-00Hx06H | -0.177 | 0.205 | 195 | -0.865 | 0.5644 |
| RESxNRES-06H | -0.287 | 0.206 | 195 | -1.397 | 0.3751 |
| RES-06Hx12H | -0.993 | 0.208 | 195 | -4.777 | **0.0001** |
| NRES-06Hx12H | -0.495 | 0.206 | 195 | -2.398 | 0.0558 |
| RESxNRE-12H | 0.211 | 0.209 | 195 | 1.010 | 0.5101 |
| RES-12Hx18H | -0.350 | 0.216 | 195 | -1.615 | 0.2878 |
| NRES-12Hx18H | -0.679 | 0.204 | 195 | -3.330 | **0.0055** |
| RESxNRES-18H | -0.118 | 0.214 | 195 | -0.554 | 0.6628 |
| RES-18Hx24H | -0.164 | 0.217 | 195 | -0.755 | 0.6017 |
| NRES-18Hx24H | -0.090 | 0.208 | 195 | -0.430 | 0.7123 |
| RESxNRES-24H | -0.044 | 0.213 | 195 | -0.207 | 0.8359 |
| RES-24Hx36H | 0.786 | 0.225 | 195 | 3.498 | **0.0046** |
| NRES-24Hx36H | 0.609 | 0.208 | 195 | 2.924 | **0.0154** |
| RESxNRES-36H | 0.221 | 0.221 | 195 | 1.000 | 0.5101 |
